# Effects of plant diversity, soil microbial diversity, and network complexity on ecosystem multifunctionality in a tropical rainforest

**DOI:** 10.3389/fpls.2023.1238056

**Published:** 2023-09-18

**Authors:** Yanxuan Chen, Xiaobo Huang, Xuedong Lang, Rong Tang, Rui Zhang, Shuaifeng Li, Jianrong Su

**Affiliations:** ^1^ Institute of Highland Forest Science, Chinese Academy of Forestry, Kunming, China; ^2^ Pu’er Forest Ecosystem Research Station, National Forestry and Grassland Administration of China, Kunming, China; ^3^ Pu’er Forest Ecosystem Observation and Research Station of Yunnan Province, Science and Technology Department of Yunnan Province, Kunming, China

**Keywords:** tropical rainforest, soil microbial diversity, plant diversity, liana species richness, ecosystem multifunctionality

## Abstract

**Introduction:**

Plant diversity and soil microbial diversity are important driving factors in sustaining ecosystem multifunctionality (EMF) in terrestrial ecosystems. However, little is known about the relative importance of plant diversity, soil microbial diversity, and soil microbial network complexity to EMF in tropical rainforests.

**Methods:**

This study took the tropical rainforest in Xishuangbanna, Yunnan Province, China as the research object, and quantified various ecosystem functions such as soil organic carbon stock, soil nutrient cycling, biomass production, and water regulation in the tropical rainforest to explore the relationship and effect of plant diversity, soil microbial diversity, soil microbial network complexity and EMF.

**Results:**

Our results exhibited that EMF decreased with increasing liana species richness, soil fungal diversity, and soil fungal network complexity, which followed a trend of initially increasing and then decreasing with soil bacterial diversity while increasing with soil bacterial network complexity. Soil microbial diversity and plant diversity primarily affected soil nutrient cycling. Additionally, liana species richness had a significant negative effect on soil organic carbon stocks. The random forest model suggested that liana species richness, soil bacterial network complexity, and soil fungal network complexity indicated more relative importance in sustaining EMF. The structural equation model revealed that soil bacterial network complexity and tree species richness displayed the significantly positive effects on EMF, while liana species richness significantly affected EMF via negative pathway. We also observed that soil microbial diversity indirectly affected EMF through soil microbial network complexity. Soil bulk density had a significant and negative effect on liana species richness, thus indirectly influencing EMF. Simultaneously, we further found that liana species richness was the main indicator of sustaining EMF in a tropical rainforest, while soil bacterial diversity was the primary driving factor.

**Discussion:**

Our findings provide new insight into the relationship between biodiversity and EMF in a tropical rainforest ecosystem and the relative contribution of plant and soil microibal diversity to ecosystem function with increasing global climate change.

## 1 Introduction

With the frequent disturbances of human activities and abnormal changes in the global climate, biodiversity is currently being lost at an unprecedented rate worldwide, which results in the loss of multiple ecosystem functions and services in the global terrestrial ecosystems (Lopez-Rojo et al., 2019; Li et al., 2023). Previous studies have mainly focused on the effects of biodiversity on individual ecosystem functions and ignored the multiple ecosystem functions provision, which displayed a biased and limited understanding of the effects of biodiversity on ecosystem functions (Li et al., 2020a; Luo et al., 2022). Previous studies have also shown that biodiversity played and important role in affecting ecosystem functions (Lefcheck et al., 2015; Yan et al., 2023), which is becoming a more prominent consideration when examining multiple ecosystem functions. Ecosystems have many functions and services, all of which may be affected by biodiversity and enhanced or inhibited by other functions (Pennekamp et al., 2018). Therefore, we need to consider the effects of biodiversity on multiple ecosystem functions simultaneously (ecosystem multifunctionality, EMF) (van der Plas et al., 2016; Garland et al., 2021).

The effect of soil microbe and plant community on EMF is a powerful tool for interpreting the relationship between biodiversity and EMF. Previous studies suggested that plant diversity and soil microbial diversity were essential to sustaining EMF (Li et al., 2020a; Wang et al., 2021), which found that EMF increased with increasing plant species richness (Maestre et al., 2012; Moi et al., 2021). As a central component of terrestrial ecosystems, soil microbial communities account for one-quarter of the Earth’s biodiversity, which played a critical role in maintaining EMF by affecting soil organic matter turnover (Bastida et al., 2016; Sokol et al., 2022). A previous study have found that soil microbial diversity drives the EMF in the terrestrial ecosystem (Delgado-Baquerizo et al., 2016). Especially, soil bacterial diversity positively promoted EMF enhancement (Giguere-Tremblay et al., 2020). However, soil fungal diversity was not significantly correlated with EMF (Jing et al., 2015; Sun et al., 2023). These suggested that there is a trade-off between different soil microbial diversity and EMF (Sun et al., 2023). Meanwhile, the relationship between plant and soil microbial diversity and EMF reveal significant differences within diverse terrestrial ecosystems. In the grassland ecosystem, EMF decreased with the loss of soil microbial diversity and the simplification of soil microbial community composition (Wagg et al., 2014; Luo et al., 2023). In the temperate forest, above-ground and subsurface biodiversity regulated EMF (Yuan et al., 2020), and environmental conditions and heterogeneity affected the stability of the ecosystem (Qiao et al., 2022). In the subtropical forest, soil microbial abundance and soil properties were positively correlated with EMF (Wang et al., 2022a), and plant functional trait diversity and structural diversity equally underpin EMF (Ouyang et al., 2023). However, the effects of soil microbial diversity and plant diversity on EMF in the tropical rainforest are poorly documented and understood.

Currently, most studies overlook the effect of soil microbial network complexity on EMF. Soil microbial network complexity affects the stability and adaptability of soil microbial communities and their functions (Chen et al., 2022). Therefore, exploring the complexity of the soil microbial networks could help us better understand the relationship between soil microbial diversity and EMF. A previous study suggested that soil microbial co-occurrence network, a key component of soil microbial diversity’s association with EMF, was a better predictor of EMF than soil microbial diversity (Chen et al., 2022). For example, ozone concentration increased the network complexity of soil bacteria and fungi, which may ultimately decrease EMF (Li et al., 2022b).

Environmental factors play a significant role in driving EMF. Soil physicochemical properties can directly or indirectly affect plant diversity and soil microbial diversity, thus altering EMF (Yang et al., 2021b). As main limiting factor for plant growth, soil moisture directly affected EMF via the processes of the plant-soil-atmosphere system (Asbjornsen et al., 2011). In addition, soil bulk density could affect the relationship between litter layer and forest floor and soil water regulation, carbon cycle and nutrient cycle (Nottingham et al., 2015).

Tropical rainforest covers only 7% of the world’s land area, which supports two-thirds of the world’s biodiversity (Couvreur et al., 2011; Chen et al., 2021). Due to climate change, shifting cultivation, and commercial logging, tropical rainforest habitats decreased rapidly and become increasingly fragmented, which may negatively affect ecosystem functions and services (Ding and Zang, 2005; Senior et al., 2018; Liu and He, 2022). Tropical rainforests have become one of the most productive but fragile ecosystems on Earth (Zhang et al., 2023). The tropical rainforest in Xishuangbanna is currently China’s largest and most biodiverse. However, its area has continuously decreased, especially with the increasing rubber price, which has led to large-scale primary forest degradation because of the conversion of primary forests to rubber plantations (Lan et al., 2017; Singh et al., 2019). This study aims to explore the relative importance of plant diversity and soil microbial diversity, soil microbial network complexity, and environmental factors in sustaining EMF in a tropical rainforest. Specifically, we explore the following three hypotheses: (1) EMF increases with increasing plant species diversity; (2) soil bacterial diversity displays more importance in sustaining EMF than soil fungal diversity; and (3) soil microbial network complexity mediates the relationship between biodiversity and EMF.

## 2 Materials and methods

### 2.1 Study area

The study area is located in Xishuangbanna, Yunnan Province, China (21°10′–22°40′ N and 99°55′–101°50′ E), belonging to the tropical humid zone south of the Tropic of Cancer (Zhai et al., 2017). The climate in this region is characterized as tropical monsoon climate, with an average annual temperature ranging from 15.1 to 21.7°C and an annual rainfall between 1200 and 2500 millimeters (mm) (Cao et al., 2006). The region is affected by the southwest monsoon throughout the year, with pronounced dry and wet seasons. The soil type is categorized as acidic brick-red soil (Ling et al., 2022). This region has the largest remaining area of tropical rainforest in China. In our study region, the main tree species in the tropical rainforest included *Parashorea chinensis*, *Cleidion brevipetiolatum*, *Phoebe lanceolata*, and *Garcinia cowa*. The shrub species included *Boehmeria glomerulifera* and *Pittosporopsis kerrii*, while the liana species included *Tetrastigma planicaule* and *Aganosma breviloba*.

### 2.2 Plot investigation and biomass measurement

From April to May 2021, we established three 1 hm^2^ forest dynamic plots in the Menglun, Shangyong, and Bubeng areas, totaling 75 sampling plots with the area of 20 m × 20 m. In each sampling plot, we identified and recorded all woody plants with a diameter at breast height (DBH) ≥ 1 cm and their tree height (m), including tree, liana, and shrub. The volume of plant branches after removing the bark was determined using the drainage method, and then the branches were dried in an oven at 103°C to a constant weight that was measured after drying. The branch density was calculated using the ratio of branch dry weight to volume. We calculated the above-ground biomass (AGB) among the woody plant species using species-specific allometric equations (Equation 1) based on wood density, DBH, and tree height (Byrnes et al., 2014; Li et al., 2020c).


(1) $$\text{AGB}=\left[0.0673\times {\left(\text{WD}\times \text{DB}{\text{H}}^{2}\times \text{H}\right)}^{0.976}\right]\times 25/1000$$

where AGB represents the above-ground biomass of the plant, WD represents wood density, DBH represents diameter at breast height, and H represents tree height.

### 2.3 Soil sampling and measurement

We collected a total of 75 soil samples. In each sample plot, we took five soil samples of about 250g from the center and four corners, with a 0–20 cm soil depth, and mixed them to form a composite soil sample. Then, we divided the composite soil sample into two parts for measuring the physicochemical properties of the soil and the soil microbial DNA, respectively. At the same sampling point, a stainless-steel cylinder with a volume of 100 cm^3^ was used to collect soil core, which were used to measure physical indicators such as soil bulk density (SBD), soil water content (SWC) and soil water-holding capacity (SWHC) (Reynolds, 1970; Wang et al., 2022b). Next, we used standard measurement methods to determine soil chemical indicators such as soil organic carbon (SOC), soil total nitrogen (TN), soil total phosphorus (TP), soil hydrolyzed nitrogen (HN), soil available phosphorus (AP), soil total potassium (TK), and soil available potassium (AK). We used the CHN analyzer (2400 II CHN Analyzer, PerkinElmer, Boston, MA, USA) to measure SOC and TN, and the molybdenum antimonial blue colorimetry and Olsen method to determine TP and AP (Bao, 2000; Li et al., 2022a). In addition, HN was determined by alkaline hydrolysis diffusion method. TK and AK were determined by atomic absorption spectrophotometry (Bao, 2000). The formula for calculating soil organic carbon stock (SOCS) (Equation 2) was used as follows:


(2) SOCS=0.1×c×d×r×(1−D)

where SOCS (t•hm^-2^) represents soil organic carbon stock, c (g•kg^-1^) represents soil organic carbon content, d (g•cm^-3^) represents soil bulk density, r (cm) represents soil layer thickness, and D represents the gravel content with a diameter greater than 2 mm, measured as a volume ratio.

### 2.4 Soil microbial diversity

We extracted DNA from 0.5 g soil samples using the FastDNA Soil DNA Isolation Kit (MP Biochemicals, Solon, OH, USA). Soil microbial diversity was analyzed using the nucleotide conserved region of rRNA. For bacteria, we used the V4 - V5 region of the highly conserved 16S gene, and for fungi, we mainly used the internal transcriptional spacer (ITS) region. The bacterial 16S rRNA was amplified with gene primers 338F (5’-ACTCCTACGGGAGGCAGCA-3’) and 806R (5’-GGACTACHVGGGTWTCTAAT-3’). The ITS of fungi was amplified by primers ITS1 (5’-CTTGGTCATTTAGAGGAAGTAA-3’) and ITS2 (5’-GCTGCGTTCTTCATCGATGC-3’). After PCR, the amplicons were extracted and purified for downstream library preparation for high-throughput sequencing. We used the Illumina HiSeq sequencing platform to analyze soil microbial diversity and constructed short fragment libraries using paired-end sequencing methods to analyze microbial species annotation and abundance. Among them, polymerase chain reaction (PCR) was performed in triplicate using 50 μl of reaction mixture containing 10 μl buffer, 0.2 μl Q5 High-Fidelity DNA polymerase, 10 μl High GC Enhancer, 1 μl Dntp, 10 μM per primer, and 60 ng genomic DNA. The PCR for fungi was performed using the Veriti Thermal cycler (Applied Biosystems). The thermal cycle conditions were 95°C for 5 min, then 95°C for 1 min, 50°C for 1 min, 72°C for 1 min, a total of 15 cycles, and finally extended for 10 min at 72°C (Li et al., 2020c). We performed amplicon sequencing using the Illumina MixSeq 2500 platform of Biomarker Technologies Corporation, Beijing, China. Low quality sequences (quality score< 20, length< 150 bp, total expected error > 0.5) were filtered after combining forward and backward reads. After sequencing, we clustered or de-noised high-quality sequences of soil bacteria and fungi and divided the bacterial and fungal communities into operational taxonomic units (OTUs) based on 97% similarity. After sequencing 75 samples of soil bacteria and fungi, we obtained 6,001,239 and 6,001,299 pairs of end-reads, respectively, from which 79,167 and 79,221 clean reads were generated. The original sequencing data can be found in the NCBI Sequence Read Archive (SRA) under the accession number SUB13691156: PRJNA995948.

### 2.5 Calculating ecosystem multifunctionality

In this study, EMF include carbon stock, nutrient cycling, water regulation and biomass production. Among them, biomass production represented the above-ground biomass (AGB) of all woody plants with DBH ≥ 1 cm. At the same time, nutrient cycling was composed of soil total nitrogen (TN), soil total phosphorus (TP), soil hydrolyzed nitrogen (HN), soil available phosphorus (AP), soil total potassium (TK), and soil available potassium (AK) in the ecosystem. Soil organic carbon stocks (SOCS) served as a proxy for carbon stock, and soil water-holding capacity (SWHC) represented water regulation.

We standardized these four ecosystem functions across 75 sampling plots using Z-score transformations. Then, the four ecosystem function scores for each sampling plots were averaged to obtain a multifunctional index (Byrnes et al., 2014; Yang et al., 2023).

### 2.6 Data analysis

All of the statistical analyses were performed using R4.2.3 (R Core Team, 2022). First, we use a multithreshold multifunctional method to evaluate the effects of plant diversity and soil microbial diversity on EMF, and analyze the trade-offs between different ecosystem functions of EMF to verify whether plant diversity and soil microbial diversity support multiple ecosystem functions at high threshold levels. First, we run this method using the “multifunc” package. The effects of plant diversity and soil microbial diversity on EMF were examined by the slope changes of curves fitted with the EMF index (threshold-based index of multifunctionality (MFt)) at different thresholds (Byrnes et al., 2014). Among them, the minimum threshold when diversity has an effect on multifunctionality (*Tmin*), the maximum threshold when diversity has an insignificant effect on multifunctionality (*Tmax*), the threshold when diversity has the greatest positive or negative effect (*Tmde*), and the threshold when diversity has the greatest effect on the number of functions that reach the threshold (*Rmde*). These four main indicators give us information on how plant diversity and soil microbial diversity affect EMF.

Next, we used the “agricolae” package to conduct ordinary least squares regression analysis to explore the relationship between plant diversity (including tree, shrub, and liana) and soil microbial diversity, soil microbial network complexity, soil water content, soil bulk density, and EMF (R Core Team, 2022). Then, we used the “linkET” package to perform correlated analyses and visualizations of the relationships between plant diversity, soil microbial diversity, soil microbial network complexity, environmental factors, and EMF. We used the ‘WGCNA’ package to construct a co-occurrence network of soil bacteria and fungi based on Spearman correlation coefficient for microbial co-occurrence network analysis. In our study, only OTUs with a relative abundance > 0.01% were used in the microbial co-occurrence network, where OTUs represented nodes and the correlations between nodes represented edges (Qiu et al., 2021). We used the “RMThreshold” package to set appropriate similarity thresholds of 0.850 and 0.540 for soil bacteria and fungi based on random matrix theory (RMT). Then, we adjusted the P-values (P< 0.05) of the correlations using the Benjamini-Hochberg false discovery rate (FDR) (Benjamini et al., 2006). We use the “*subgraph*” function in the “igraph” package to extract the subnetwork properties of each soil sample from the constructed network (Yang et al., 2022). Then, the soil microbial network was visualized using the Gephi platform (Xue et al., 2017). Soil bacterial and fungal network complexity was calculated using the topological features of the network, including the number of nodes and edges, average degree, clustering coefficient, average path length, network diameter, and graph density (Jiao et al., 2022). We also checked the dominant taxonomic groups at the phylum level (Qiu et al., 2021).

Next, we use multidimensional scales (MDS) to calculate topological parameters of soil microbial network complexity. Before calculation, the average path length and diameter are processed using the reciprocal of the variable (X-1) (Jiao et al., 2022). Then, before statistical analysis, all data were log10-transformed for normality. OTUs represented the alpha diversity index of soil bacteria and fungi, and plant diversity is mainly represented by plant species richness, included the number of tree, shrub, and liana species in each sampling plot. The effects of plant diveristy, soil microbial diversity, soil microbial network complexity, and environmental factors on EMF were assessed using ordinary least squares linear regression.

Finally, we use “lavaan” (Rosseel, 2012) and “randomForest” packages to analyze SEM and random forest model respectively. We used random forest model mean square error (MSE) percentages to assess the relative importance of plant diversity, soil microbial diversity, soil microbial network complexity, and environmental factors to EMF (Jiao et al., 2022). Next, we used structural equation modeling (SEM) to assess the direct and indirect associations between plant diversity, soil microbial diversity, soil microbial network complexity, environmental factors, and EMF. The effects of different variables on EMF and multiple ecosystem functions were determined by path standardization coefficients and correlation P-values. We use Chi-square tests, goodness of fit index (GFI), and approximate root-mean-square error (RMSEA) to evaluate the fit of the model. The results showed that P > 0.05, GFI = 1, RMSEA< 0.05 by chi-square test (Rosseel, 2012), indicating that the structural equation model was trustworthy.

## 3 Results

### 3.1 Effects of plant diversity and soil microbial diversity on EMF

**Figure 1:**
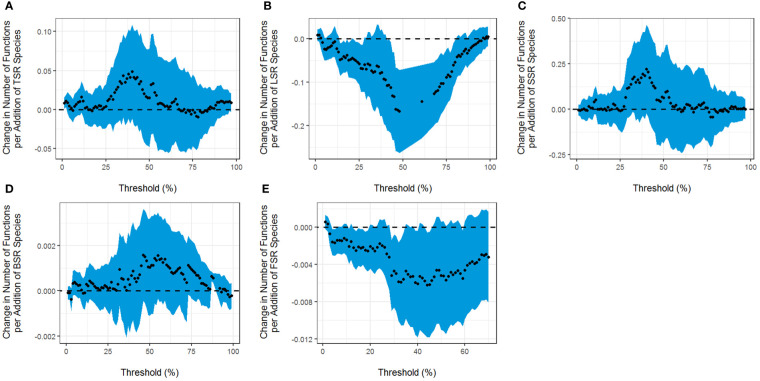
Correspondence between the slope of the relationship between the threshold and the **(A)** tree species richness (TSR), **(B)** liana species richness (LSR), **(C)** shrub species richness (SSR), **(D)** soil bacterial diversity (BSR), **(E)** soil fungal diversity (FSR), and the number of functions that reached the threshold. The dots represent the fitted values, and the shaded areas represent ± 1 confidence interval.

According to the results obtained from the multithreshold method, we can conclude that TSR has a significant positive correlation with EMF in the 1% to 3% threshold range (**Figure 1A**). LSR has a significant negative effect on EMF, with a minimum threshold of 13% and a maximum threshold of 81%. When the threshold reached 48%, the effect on EMF was the greatest, and the maximum effect (*Rmde*) of LSR was 0.1668, meaning that an increase of one liana species reduced 0.1668 functional scores (**Figure 1B**). SSR has no significant effect on EMF (**Figure 1C**). In addition, BSR has a significant positive effect on EMF, with a minimum threshold of 86% and a maximum threshold of 92% (**Figure 1D**). In the 3% to 60% threshold range, FSR has a significant negative effect on EMF (**Figure 1E**).

### 3.2 Relationship between EMF and plant diversity, soil microbial diversity, soil microbial network complexity, and environmental factors

**Figure 2:**
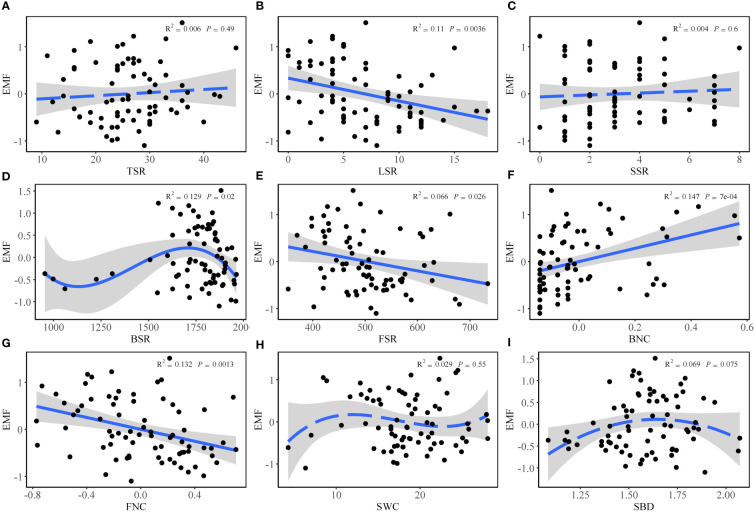
Relationship between EMF and **(A)** tree species richness (TSR), **(B)** liana species richness (LSR), **(C)** shrub species richness (SSR), **(D)** soil bacterial diversity (BSR), **(E)** soil fungal diversity (FSR), **(F)** soil bacterial network complexity (BNC), **(G)** soil fungal network complexity (FNC), **(H)** soil water content (SWC), and **(I)** soil bulk density (SBD) based on ordinary least squares linear regression analysis.

Based on ordinary least squares linear regression analysis, EMF decreased with increasing LSR (**Figure 2B**), BSR (**Figure 2D**), FSR (**Figure 2E**) and FNC (**Figure 2G**). However, EMF showed an increasing trend with the increase in BNC (**Figure 2F**). Additionally, there was no significant regression relationship between EMF and TSR (**Figure 2A**), SSR (**Figure 2C**), SWC (**Figure 2H**), and SBD (**Figure 2I**).

**Figure 3:**
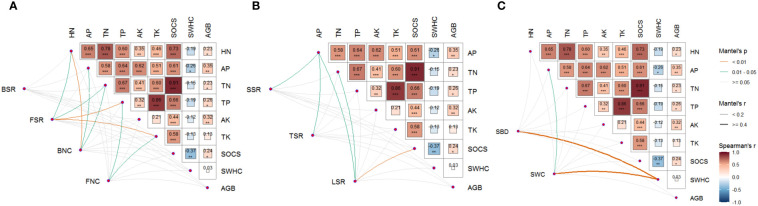
Relationship between **(A)** soil microbial diversity and network complexity, **(B)** plant diversity, and **(C)** environmental factors and multiple ecological functions. BSR, soil bacterial diversity; FSR, soil fungal diversity; BNC, soil bacterial network complexity; FNC, soil fungal network complexity; SSR, shrub species richness; TSR, tree species richness; LSR, liana species richness; SBD, soil bulk density; SWC, soil water content; TN, soil total nitrogen; TP, soil total phosphorus; HN, soil available nitrogen; AP, soil available phosphorus; TK, soil total potassium; AK, soil available potassium; SWHC, soil water-holding capacity; SOCS, soil organic carbon stock; GB, above-ground biomass.

Further, combined heatmap analysis showed that FSR, FNC, and BNC had significant positive effects on nutrient cycling function, and the affected soil nutrients mainly included TN, TP, TK, HN, and AP (**Figure 3A**). SSR, TSR, and LSR mainly have significant positive effects on AP, and LSR also has significant positive effects on TN and SOCS (**Figure 3B**). SBD and SWC have significant positive effects on SWHC, and SWC also has a positive effect on AP (**Figure 3C**).

### 3.3 Soil microbial network complexity and dominant phyla

**Figure 4:**
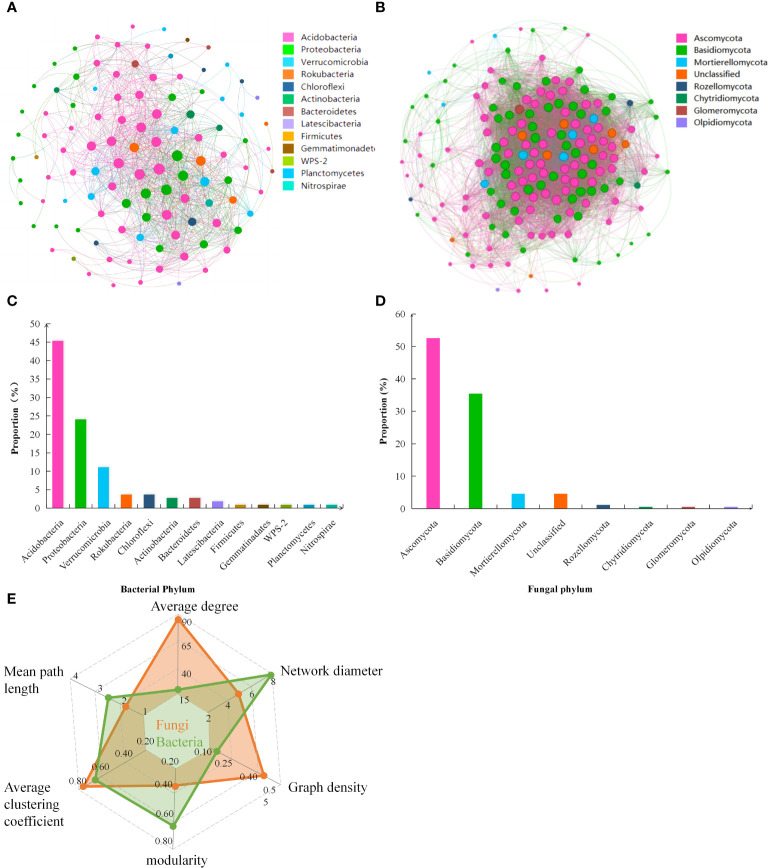
Co-occurrence networks of **(A)** soil bacteria and **(B)** soil fungi, and the proportion of dominant phylum of **(C)** soil bacteria and **(D)** soil fungi to total phyla abundance; **(E)** The radar chart represents the relevant topological parameters of the networks.

Soil bacterial and fungal communities in the soil exhibited differential patterns of symbiosis, but both soil bacterial and fungal co-occurrence networks demonstrated high complexity. Based on the topological parameters of the soil microbial networks (**Figure 4E**), the average degree of soil bacteria network was 16.889. The network diameter was 8, with a graph density of 0.158. The modularity was 0.599, and the average clustering coefficient of the nodes was 0.661, with an average path length of 2.647. On the other hand, the average degree of soil fungi network was 88.041. The network diameter was 5, with a graph density of 0.454. The modularity was 0.359, and the average clustering coefficient of the nodes was 0.786, with an average path length of 1.713.

**Figure 5:**
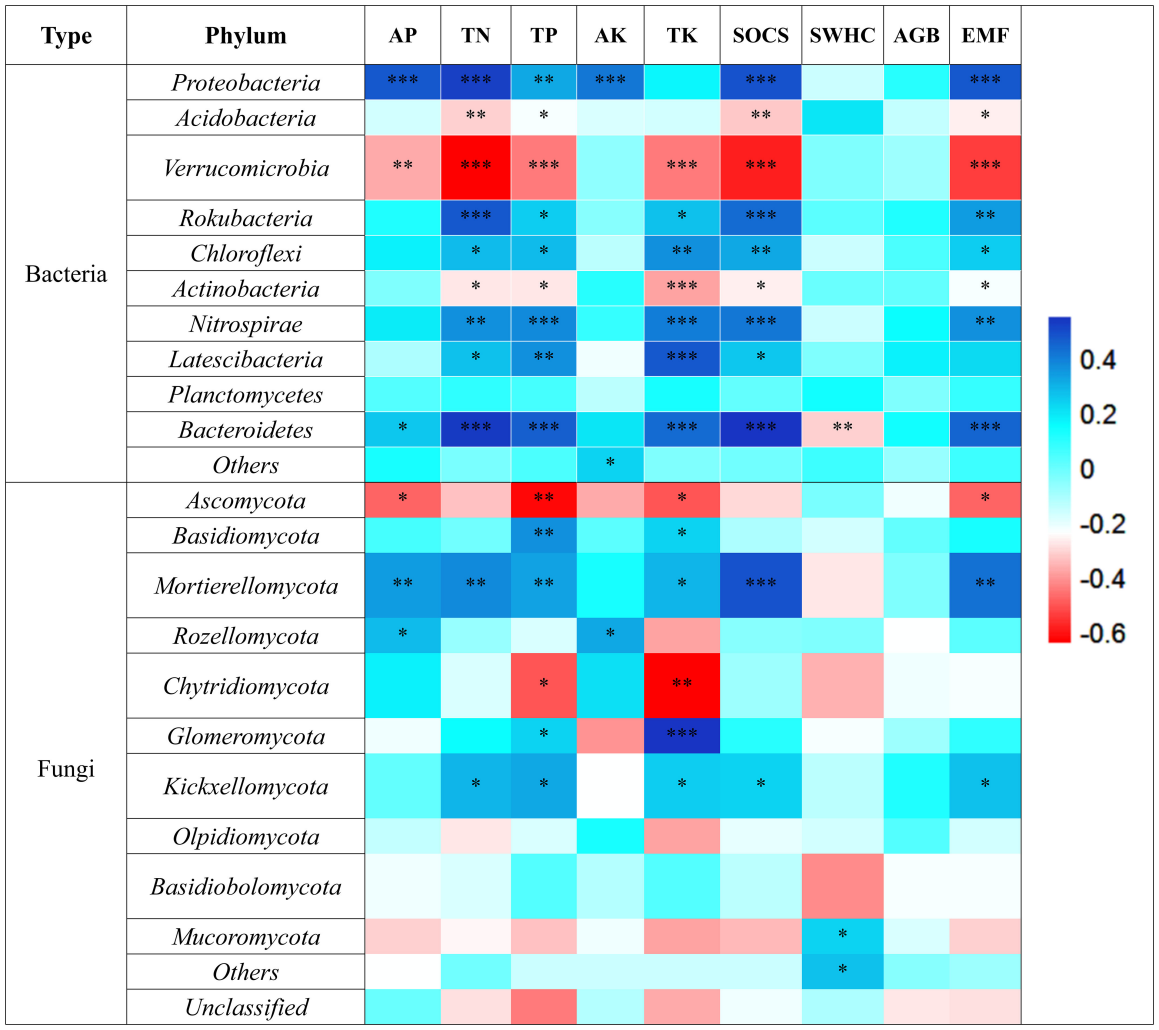
Relationship between soil bacterial dominant groups, soil fungal dominant groups and EMF *, p< 0.05; **, p< 0.01; ***, p< 0.001.

At the phylum level, the dominant groups of soil bacteria and their proportion in the total phylum abundance (**Figures 4A, C**), were *Acidobacteria* (45.37%), *Proteobacteria* (24.07%), and *Verrucomicrobia* (11.11%), among others. The dominant groups of soil fungi were *Ascomycota* (52.57%) and *Basidiomycota* (35.43%) (**Figures 4B, D**). Among the dominant bacterial groups, *Acidobacteria* and *Verrucomicrobia* showed a significant negative correlation with EMF, mainly influencing the soil nutrient cycling function of TN, TP, TK, and SOCS. On the other hand, *Proteobacteria* showed a significant positive correlation with EMF, positively impacting the nutrient cycling of TN, TP, and TK and carbon stock function (**Figure 5**). Among the dominant fungal groups, *Ascomycota* had a significant negative correlation with EMF, primarily affecting nutrient cycling function of AP, TP, and TK. Conversely, *Basidiomycota* were positively correlated with EMF, mainly influencing nutrient cycling function of TP, TK, and carbon stock function (**Figure 5**).

### 3.4 Relative importance of plant diversity, soil microbial diversity, soil microbial network complexity, and environmental factors on EMF

**Figure 6:**
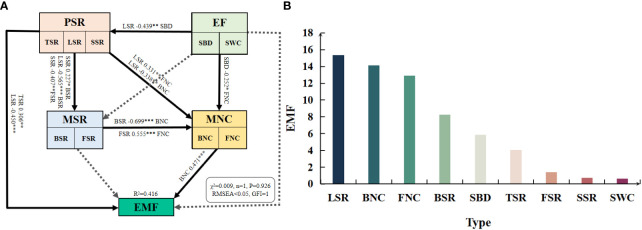
Effects and significance of plant diversity, soil microbial diversity, soil microbial network complexity and environmental factors on EMF. PSR: plant species richness included tree species richness (TSR), liana species richness (LSR), and shrub species richness (SSR); EF: environmental factors included soil water content (SWC) and soil bulk density (SBD); MSR: soil microbial diversity included soil bacterial diversity (BSR) and soil fungal diversity (FSR); MNC: soil microbial network complexity included soil bacterial network complexity (BNC) and soil fungal network complexity (FNC). Solid lines and standardized path coefficients indicate significant differences (P< 0.05), while dashed lines indicate no significant differences. *, p< 0.05; **, p< 0.01; ***, p< 0.001; RMSEA (Root Mean Square Error of Approximation) < 0.05 and GFI (Goodness of Fit Index) ≥ 0.9, indicates that the model fits reasonably.

According to the random forest model (**Figure 6B**), LSR, BNC, and FNC ranked the top three in the importance of contributing EMF, with importance scores of 15.37, 14.13, and 12.91, respectively, while FSR, SSR, and SWC ranked the least in the importance of EMF, with scores of 1.41, 0.74, and 0.64, respectively.

The standardized effects of plant diveristy, environmental factors, soil microbial diversity, and soil microbial network complexity on EMF were obtained through structural equation modeling (SEM) (**Figure 6A**). Chi-square test showed no significant P-value (0.926 > 0.05), indicating that there was no significant difference between the implied covariance matrix of the model and the observed covariance matrix (P< 0.05), which confirmed the validity of the structural equation model. The results demonstrated that BNC has a highly significant direct positive effect on EMF (path standardized coefficient = 0.471, P< 0.001). TSR also has a significant direct positive effect on EMF (path standardized coefficient = 0.306, P< 0.01), while LSR has a highly significant direct negative effect on EMF (path standardized coefficient = -0.450, P< 0.001). Furthermore, MSR primarily exerted an indirect effect on EMF by influencing MNC, while environmental factors (EF) had significant negative effects on LSR and FNC, thus indirectly affecting EMF.

## 4 Discussion

Our results reveal a positive correlation between tree species richness and EMF. Our findings revealed a positive correlation between tree species richness and EMF, which is consistent with most previous studies showing that EMF increases as tree species richness increases (Eisenhauer et al., 2018; Moi et al., 2021). At the same time, this also confirms our first hypothesis. However, it is worth noting that the increase in liana species richness has a significant negative effect on EMF. There are several possible explanations for this phenomenon. First, tropical rainforests have been shown to contain more diverse plant species and complex community structures but lack dominant species (Purves and Turnbull, 2010). Therefore, the increase of liana species richness will affect the carbon stock function and nutrient cycling function of tropical rainforests, and significantly influences the dynamic development of tropical rainforests and adversely affect the reproduction, growth, and survival of tree species (van der Heijden and Phillips, 2009; Schnitzer et al., 2011). Secondly, most liana species were considered pioneer plants with high light requirements. They often compete with certain tree species for light to affect inter-species coexistence and species diversity. This competition limited the survival of understory plants and reduced the overall productivity and net primary productivity of trees (Rodriguez-Ronderos et al., 2016; Meunier et al., 2021; Meunier et al., 2022), thereby reducing ecosystem productivity and multifunctionality. Therefore, we suggest that liana species richness may be the main indicator of EMF in the recovery of tropical rainforests.

Soil microbial diversity was a driving factor in sustaining EMF, and soil bacteria and soil fungi played different roles in this process. Our study found that soil bacterial diversity was the main driver of EMF in tropical rainforests. Soil bacterial diversity has a significant positive effect on EMF, but when it exceeded a certain threshold, soil bacterial diversity has a negative effect on EMF. In contrast, soil fungal diversity was significantly negatively correlated with EMF. This finding was consistent with previous research indicating that EMF was significantly positively correlated with soil bacterial diversity rather than soil fungal diversity (Sun et al., 2023). This finding also supports our second hypothesis. This relationship may be due to the greater flexibility of soil bacteria in terms of resource requirements and physiological capabilities (He et al., 2009). Increasing the diversity of soil bacteria can alter the physicochemical properties of the rhizosphere, facilitating the absorption of nutrient and water by plant roots (Li et al., 2020d) and enhancing the synergistic interactions between plants and microorganisms.

In addition, soil bacterial diversity has been shown to actively participate in the cycling of organic compounds produced by plants, fungi, and insects (Kalam et al., 2020), thereby promoting plant growth and increasing EMF. However, with the increase of soil bacterial diversity, niche differentiation occurred in the forest soil microenvironment, which limited the survival space of soil microorganisms and intensified the competition for available resource among soil microorganisms. This interaction hinders the growth of EMF beyond a certain threshold (Chen et al., 2022). The increase in soil fungal diversity accelerates the decomposition of litter in tropical rainforests, potentially leading to an increase in pathogenic fungi and saprophytic fungi and a decrease in mutualistic fungi, thus negatively affecting the survival and growth of forest plants (Li et al., 2022c). Furthermore, soil fungi have been observed to negatively impact plant growth and EMF through symbiotic and pathogenic interactions, influencing nutrient availability and cycling (Frac et al., 2018).

Currently, soil microbial network complexity is being investigated as a driving mechanism for EMF. Previous studies found a strong negative correlation between soil microbial network complexity and EMF (Li et al., 2022b), corroborating the negative correlation between soil fungal network complexity and EMF observed in our study. However, we also found a significant positive correlation between soil bacterial network complexity and EMF. The possible reason for this is that soil microbial keystone species regulate related microbial structures and EMF (Yang et al., 2021a). For example, in a dominant bacterial community, we identified *Acidobacteria*, which have been found to interact positively with plants and are considered plant growth-promoting bacteria. *Acidobacteria* possess beneficial genes that contribute to their survival and competitive colonization in the rhizosphere, establish a beneficial relationship with plants, and promote soil nutrient cycling and carbon stock, thereby facilitating plant growth and enhancing EMF (Kielak et al., 2016; Kalam et al., 2020). Other studies have shown that the abundance of *Ascomycota* is significantly higher in tropical forest soils than in other forest ecosystems (Tedersoo et al., 2014). This increased abundance of *Ascomycota* may be attributed to the warm and humid climate, abundant biological resources, and higher litter decomposition rates in tropical forests. *Ascomycota* can degrade litter and other organic substrates and is a typical fungal phylum involved in decaying processes (Tedersoo et al., 2014; Li et al., 2022b). This means that increase in the abundance of *Ascomycota* has negative effects on soil nutrient cycling and carbon stock, which are not conducive to maintaining biodiversity and EMF growth. This also provides strong evidence for our conclusion and third hypothesis.

## 5 Conclusions

Our study demonstrates that liana species richness played an important role in changes of EMF as an indicator, while soil bacterial diversity was a key driving factor in sustaining EMF. Soil bacterial diversity indirectly affected EMF via soil bacterial network complexity. Furthermore, soil fungal diversity, soil fungal network complexity, and soil bacterial network complexity played more critical roles in soil nutrient cycling, while liana species richness primarily affected soil nutrient cycling and carbon stocks. Therefore, liana species richness and soil microbial network complexity played a crucial role in EMF within a tropical rainforest. In the restoration of tropical rainforest, our findings suggest promoting the recovery and improvement of EMF in tropical rainforest by appropriately reducing the type and liana. At the same time, we also suggest reducing the diversity of soil fungi and increasing the network complexity of soil bacterial dominant groups to promote the restoration of tropical rainforest and the promotion of EMF. When evaluating EMF through the lens of tropical rainforest ecosystem conservation and sustainable management, the effects and interactions of above-ground and below-ground biodiversity should be considered simultaneously.

## Data availability statement

The datasets presented in this study can be found in online repositories. The names of the repository/repositories and accession number(s) can be found below: BioProject, PRJNA995948.

## Author contributions

CY, LS and SJ conceived the concept for this study and designed the methodology. LS, HX, TR, LX, and ZR collected the data. LS contributed to revisions. CY analysed the data and wrote the manuscript. All authors contributed to the article and approved the submitted version.
